# COSMIC: a curated database of somatic variants and clinical data for cancer

**DOI:** 10.1093/nar/gkad986

**Published:** 2023-11-01

**Authors:** Zbyslaw Sondka, Nidhi Bindal Dhir, Denise Carvalho-Silva, Steven Jupe, Madhumita, Karen McLaren, Mike Starkey, Sari Ward, Jennifer Wilding, Madiha Ahmed, Joanna Argasinska, David Beare, Manpreet Singh Chawla, Stephen Duke, Ilaria Fasanella, Avirup Guha Neogi, Susan Haller, Balazs Hetenyi, Leonie Hodges, Alex Holmes, Rachel Lyne, Thomas Maurel, Sumodh Nair, Helder Pedro, Amaia Sangrador-Vegas, Helen Schuilenburg, Zoe Sheard, Siew Yit Yong, Jon Teague

**Affiliations:** Wellcome Sanger Institute, Wellcome Genome Campus, Hinxton, Cambridge CB10 1SA, UK; Wellcome Sanger Institute, Wellcome Genome Campus, Hinxton, Cambridge CB10 1SA, UK; Wellcome Sanger Institute, Wellcome Genome Campus, Hinxton, Cambridge CB10 1SA, UK; Wellcome Sanger Institute, Wellcome Genome Campus, Hinxton, Cambridge CB10 1SA, UK; Wellcome Sanger Institute, Wellcome Genome Campus, Hinxton, Cambridge CB10 1SA, UK; Wellcome Sanger Institute, Wellcome Genome Campus, Hinxton, Cambridge CB10 1SA, UK; Wellcome Sanger Institute, Wellcome Genome Campus, Hinxton, Cambridge CB10 1SA, UK; Wellcome Sanger Institute, Wellcome Genome Campus, Hinxton, Cambridge CB10 1SA, UK; Wellcome Sanger Institute, Wellcome Genome Campus, Hinxton, Cambridge CB10 1SA, UK; Wellcome Sanger Institute, Wellcome Genome Campus, Hinxton, Cambridge CB10 1SA, UK; Wellcome Sanger Institute, Wellcome Genome Campus, Hinxton, Cambridge CB10 1SA, UK; Wellcome Sanger Institute, Wellcome Genome Campus, Hinxton, Cambridge CB10 1SA, UK; Wellcome Sanger Institute, Wellcome Genome Campus, Hinxton, Cambridge CB10 1SA, UK; Wellcome Sanger Institute, Wellcome Genome Campus, Hinxton, Cambridge CB10 1SA, UK; Wellcome Sanger Institute, Wellcome Genome Campus, Hinxton, Cambridge CB10 1SA, UK; Wellcome Sanger Institute, Wellcome Genome Campus, Hinxton, Cambridge CB10 1SA, UK; Wellcome Sanger Institute, Wellcome Genome Campus, Hinxton, Cambridge CB10 1SA, UK; Wellcome Sanger Institute, Wellcome Genome Campus, Hinxton, Cambridge CB10 1SA, UK; Wellcome Sanger Institute, Wellcome Genome Campus, Hinxton, Cambridge CB10 1SA, UK; Wellcome Sanger Institute, Wellcome Genome Campus, Hinxton, Cambridge CB10 1SA, UK; Wellcome Sanger Institute, Wellcome Genome Campus, Hinxton, Cambridge CB10 1SA, UK; Wellcome Sanger Institute, Wellcome Genome Campus, Hinxton, Cambridge CB10 1SA, UK; Wellcome Sanger Institute, Wellcome Genome Campus, Hinxton, Cambridge CB10 1SA, UK; Wellcome Sanger Institute, Wellcome Genome Campus, Hinxton, Cambridge CB10 1SA, UK; Wellcome Sanger Institute, Wellcome Genome Campus, Hinxton, Cambridge CB10 1SA, UK; Wellcome Sanger Institute, Wellcome Genome Campus, Hinxton, Cambridge CB10 1SA, UK; Wellcome Sanger Institute, Wellcome Genome Campus, Hinxton, Cambridge CB10 1SA, UK; Wellcome Sanger Institute, Wellcome Genome Campus, Hinxton, Cambridge CB10 1SA, UK; Wellcome Sanger Institute, Wellcome Genome Campus, Hinxton, Cambridge CB10 1SA, UK

## Abstract

The Catalogue Of Somatic Mutations In Cancer (COSMIC), https://cancer.sanger.ac.uk/cosmic, is an expert-curated knowledgebase providing data on somatic variants in cancer, supported by a comprehensive suite of tools for interpreting genomic data, discerning the impact of somatic alterations on disease, and facilitating translational research. The catalogue is accessed and used by thousands of cancer researchers and clinicians daily, allowing them to quickly access information from an immense pool of data curated from over 29 thousand scientific publications and large studies. Within the last 4 years, COSMIC has substantially expanded its utility by adding new resources: the Mutational Signatures catalogue, the Cancer Mutation Census, and Actionability. To improve data accessibility and interoperability, somatic variants have received stable genomic identifiers that are associated with their genomic coordinates in GRCh37 and GRCh38, and new export files with reduced data redundancy have been made available for download.

## Graphical Abstract

**Graphical Abstract:**
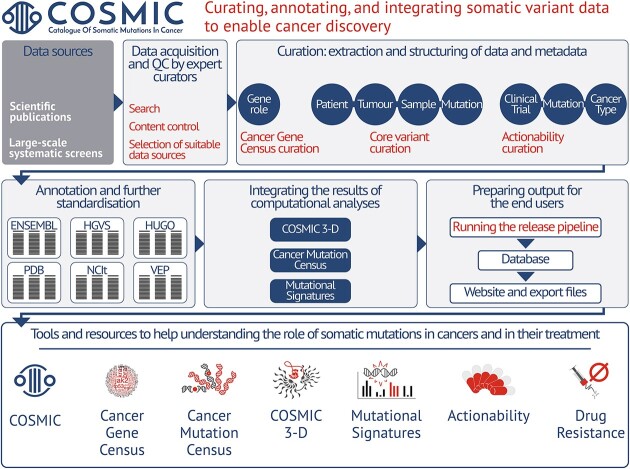

## Introduction

Characterising the genomic landscape of somatic mutations in cancer is a challenge that has been approached from several different directions. It includes carrying out large sequencing projects like TCGA (1), which characterized over 20 000 primary cancer and matched normal samples spanning 33 cancer types. An extension of this work was the ICGC/TCGA Pan-Cancer Analysis of Whole Genomes project (PCAWG) (2), which entailed the whole genome analysis of 2658 tumours and matched normal tissues, representing 38 cancer types and seeking to identify common patterns of mutation. An example of a more recent initiative is AACR Project GENIE (3), which is a publicly accessible cancer registry of real-world clinico-genomic data assembled through data sharing between 19 leading international cancer centres.

COSMIC integrates somatic data from multiple sources published around the world and allows researchers to access and scrutinise information about somatic mutations and their impact in cancer. Over the past two decades, COSMIC has been diligently collecting, cleaning, and organising genomic data and associated metadata from cancer studies published in scientific literature and various bioinformatics sources. This data is then translated into a standardised format, integrated, and made available to the research community through well-structured datasets and user-friendly data exploration websites and tools (4,5). COSMIC aligns with the mission and values of the Wellcome Sanger Institute that are dedicated to advancing our understanding of human biology and improving human health. By collating and disseminating this wealth of cancer-related data, COSMIC empowers scientists worldwide to leverage existing research findings for the benefit of future studies and, ultimately, cancer patients.

Started in 2004 as a census of somatic mutations in four cancer genes (6), COSMIC has broadened its approach to cover the entire human genome and all types of cancer. Due to the huge complexity of acquired data, numerous ways of reporting it in publications, and a vast number of data classification standards and formats, data curation in COSMIC is performed by an in-house team of doctoral level curators with developed expertise in cancer genetics, assuring high accuracy, quality, and biological relevance of the datasets.

**Figure 1.**
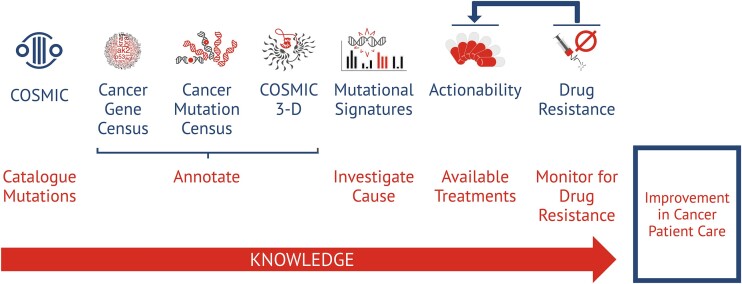
COSMIC’s 7 key resources for understanding cancer and improving cancer patient care. The main catalogue of somatic mutations is supported by further six resources that together lay additional layers of knowledge helping to interpret the impact of somatic mutations on cancer development and presenting available therapeutic options.

Somatic mutations and their impact on cancer development remain central to COSMIC. Furthermore, the catalogue of genetic alterations is supported by data from other omics analyses, including gene expression and DNA methylation variants. In addition to the main catalogue of somatic mutations, a further 6 accompanying resources focus on different aspects of oncology (Figure 1). The Cancer Gene Census (CGC) (7) and Cancer Mutation Census (CMC) provide additional annotations regarding the roles of genes and mutations in oncogenesis, which are based on a defined set of rules and sufficient evidence obtained through dedicated literature curation and analysis of the content of the core catalogue. COSMIC 3-D visualises mutation prevalence in the context of the protein structure. Mutational Signatures explore the molecular origins of mutations at genome level. Finally, Actionability and the catalogue of mutations causing drug resistance address the availability of therapeutic options for cancer and how their efficacy depends on the genetic profile of the tumour. Jointly, these complementary resources are intended to add layers of additional knowledge, show the biological impact of somatic mutations in the context of the broader spectrum of processes driving cancer, and inform about ways of targeting molecular alterations in clinical practice.

These curation efforts have resulted in a FAIR-compliant (8) resource that is available through a dedicated website (https://cancer.sanger.ac.uk/cosmic) and as set of data export files. The resource brings together data and metadata from more than 1.5 million patient samples and over 1000 cancer cell lines, acquired from over 29000 scientific publications and expanded by open-access data from large studies such as TCGA (1) and ICGC (9).

## Expert curation

**Figure 2.**
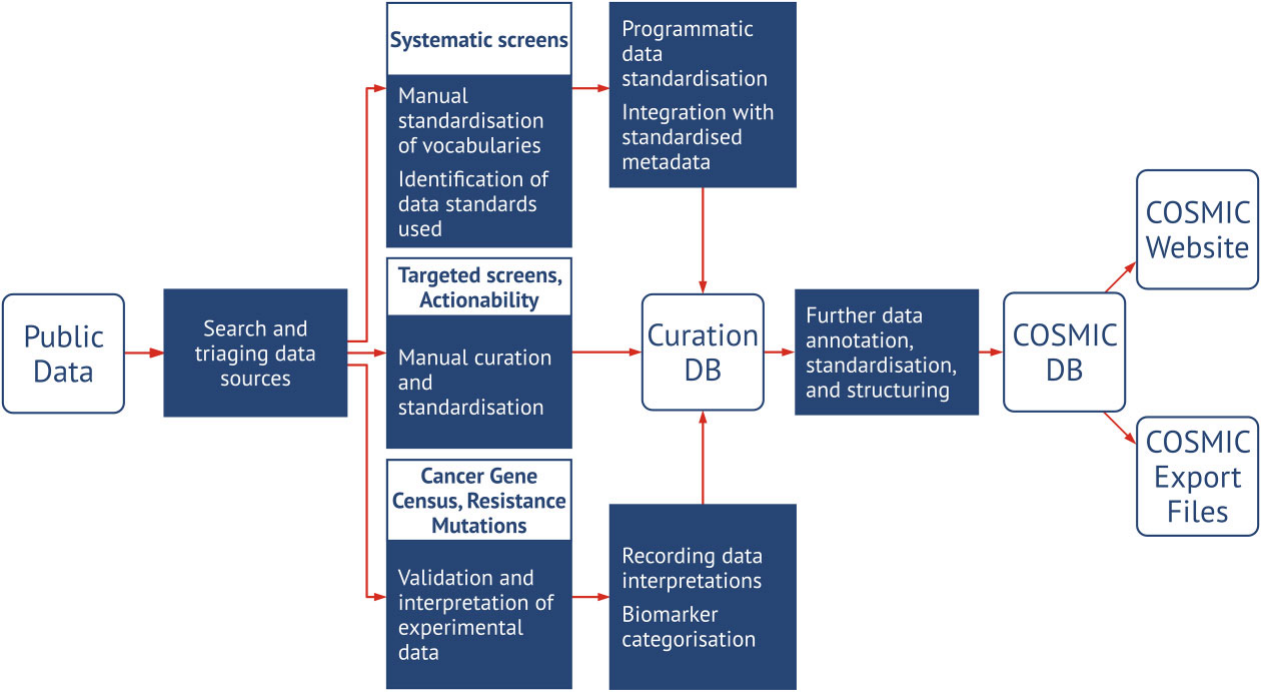
COSMIC data curation flowchart. Depending on the data source and curation objectives, there are three main curation paths in COSMIC. Somatic variant and Actionability curation always require manual standardisation of metadata to COSMIC nomenclatures. Curation of variant data is performed either in a semi-automated way for the systematic screens or manually for targeted screens. Resources such as the Cancer Gene Census, Hallmarks of Cancer or Resistance Mutations require thorough validation and interpretation of experimental data from several sources. These interpretations form the evidence base for categorisation of biomarkers reported to the users e.g. classification of a gene as tumour suppressor.

COSMIC’s workflows to manually curate cancer genetic data have been built to deliver high-quality, biologically and clinically-relevant data to the research community. Different data sources and types of curated data require different approaches (Figure 2). However, in each case there are common core elements. Firstly, the source of the information is identified from the peer reviewed literature or bioinformatic resources, and checked for the quality and relevance of the content. To enable meaningful analysis by end users, data need to be adequately and transparently categorised. This is achieved by combining the use of controlled vocabularies that label data and a database schema that is able to represent these vocabularies. Before data extraction, all curated features and terms are converted to vocabularies, ontologies and data conventions used by COSMIC. Genes, variants, and transcripts use external vocabularies and ontologies—HGNC (10), Sequence Ontology (11) and HGVS (12), or Ensembl (13), respectively. For interoperability, all COSMIC disease classifications have been mapped to the NCI thesaurus ontology (14) and these mappings can be downloaded from the COSMIC website.

Acquiring the data itself is the final stage of curation. It is particularly challenging in the case of targeted screens (see glossary), where typically a combination of information from the manuscript, its figures, and supplementary data has to be extracted and meaningfully integrated. The resulting normalised structured data and metadata are then uploaded to the curation database, which is a data source for a set of processes to create the outputs on the website and the download files for subsequent COSMIC releases.

Curation of somatic variants is prioritised for cancer driving genes identified by the Cancer Gene Census (7). When a new gene is selected for exhaustive curation, curators start by identifying publications containing data on somatic alterations as well as demographic, clinical and technical details about patients, tumours and samples. Variants are always assigned to a sample; publications not reporting mutations at a sample resolution cannot be curated.

**Figure 3.**
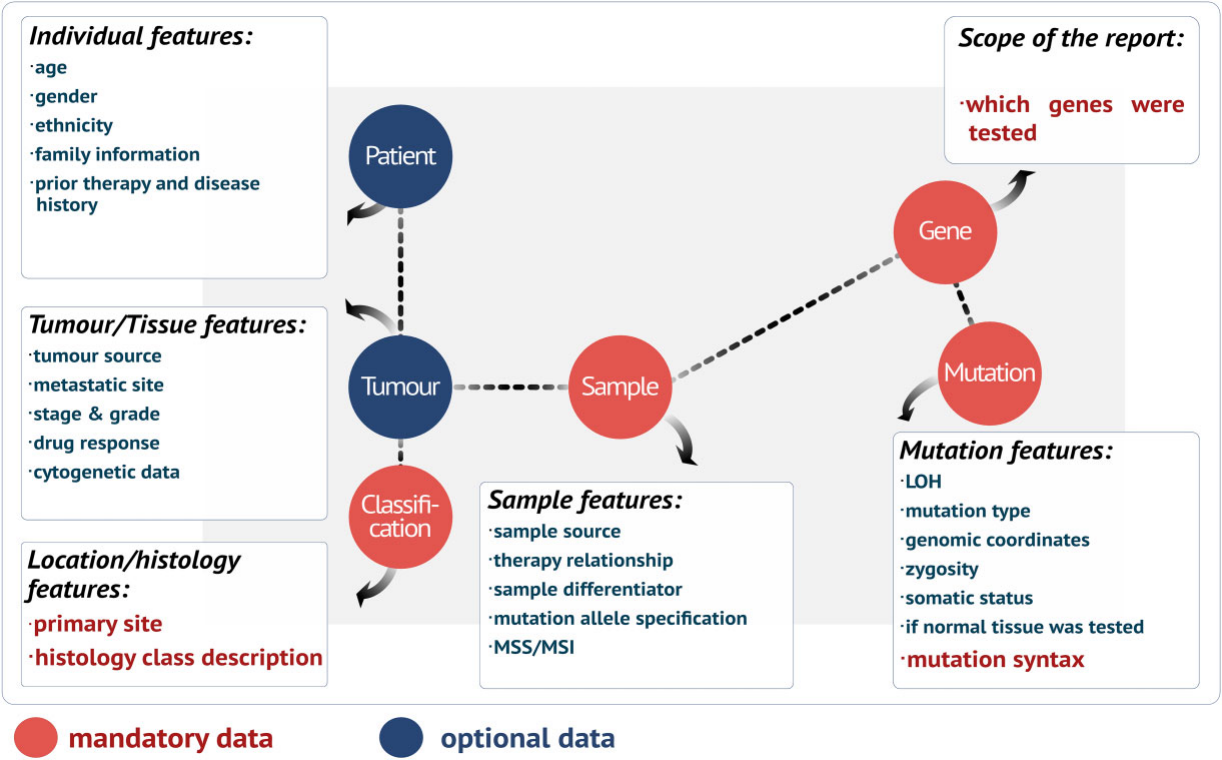
Data and metadata included in COSMIC somatic variant curation.

The minimum unit of curation is: a genetic variant, tumour type and the scope of the study, i.e. which genes were tested (Figure 3). In addition, whenever reported by the publication, other clinical features for the patient are curated e.g. age, gender, ethnicity, therapeutic history, family history of cancer or exposure to DNA-damaging agents. At the tumour level, we extract information on cancer stage and grade, metastases, drug response and therapy relationship, i.e. if a sample was collected prior to, during, or post-therapy.

## COSMIC content

While the catalogue of somatic mutations is COSMIC’s core resource, the portfolio has been expanded to help users interpret genomic data, understand the impact of somatic alterations on disease, and support translational research. With the Cancer Gene Census, COSMIC has pioneered knowledge curation on a genomic scale. Over recent years, COSMIC has added several similar knowledge catalogues that address different points in the continuum of cancer treatment: from diagnosis and understanding the causes of the disease, to prognosis and therapy choices, including the development of drug resistance to targeted drugs.

### Major changes since COSMIC release version 86

Since 2018 (5), COSMIC has grown in content and scope, with the addition of new features: Cancer Mutation Census (CMC), Actionability and a catalogue of Mutational Signatures with a web-based annotation tool (see below). Other updates to COSMIC include IT developments resulting in improved data quality control, interoperability and accessibility, which allow us to move towards becoming a FAIR-compliant resource.

### Growth of the catalogue of somatic mutations

Since v86, COSMIC has expanded its somatic catalogue with mutations curated from an additional 1981 studies containing data from 27147 cancer samples. The size of the dataset in v98, released in May 2023, is summarised in Table 1. We prioritise high quality data available in peer-reviewed publications and for each new release (currently twice a year) we may have a different curation focus whether it's curation of newly identified CGC genes or building datasets focused on rarer forms of cancer. Since our last NAR database issue publication (5), our curation initiatives have focused on mesothelioma (v87), cholangiocarcinoma (v89), testicular and other male cancers, as well as breast implant-associated anaplastic large cell lymphoma (BIA-ALCL) (v91), rare lung cancers and rare pancreatic cancers (v94), rare cancers of the female genital tract and breast, including ovarian germ cell tumours, uterine Mullerian tumours and breast adenomyoepithelioma (v95), rare head and neck cancers (v96), haematological neoplasms (v97), and rare skin tumours (v98). More details are available at COSMIC release notes website (https://cancer.sanger.ac.uk/cosmic/release_notes).

**Table 1.** COSMIC in numbers. Summary statistics of the content of the core COSMIC database (v98)

Total genomic variants (COSV)	23 854 105
Genomic mutations within exons (coding variants)	5 078 567
Genomic non-coding variants	16 304 701
Genomic mutations within intronic and other intragenic regions	8 878 333
Samples	1 520 321
Gene fusions	19 428
Whole genome screen samples	42 519
Copy number variants	1 207 190
Gene expression variants	9 215 470
Differentially methylated CpGs	7 9304 89

### New data integration and annotation system: genomic coordinates for each mutation

To ensure standardisation and interoperability across the entire dataset, the COSMIC database has undergone an extensive update, which has substantially improved the identification of unique variants defined at the genome, transcript, or protein level. We have introduced a new stable genomic Identifier (COSV) that indicates the position of the variant in the genome, allowing variants to be mapped between human genome assemblies, as well as between various transcriptome assemblies. The new identifier has replaced previously used transcript-level (COSM) and non-coding genomic (COSN) identifiers. However, to secure backwards data compatibility, the legacy COSM and COSN identifiers are still included in COSMIC variant reporting and interoperable with COSV identifiers.

All the transcripts and proteins are only from the high quality GENCODE basic set (GENCODE v28) (15). To ensure standardisation and uniformity in the dataset, all the variants with known genomic coordinates are mapped across transcripts and genes using the Ensembl's Variant Effect Predictor (VEP) (16), improving compliance with current HGVS standards (12) and interoperability with other resources.

### Changes to curation database and new data export files

As part of COSMIC’s database restructuring, the curated data was remodelled into a new relational database, to ensure the data is more interconnected, standardised and streamlined. This improved database was used to produce a new set of downloadable export files and download website in v98. The new files both assure improved interoperability of data by making each file more interconnected with internal and external identifiers, and are characterised by reduced data redundancy across all files. All major data points are linked with 10 COSMIC identifiers, which can be used to effectively join the data content from multiple files: COSMIC Phenotype Id (COSO), COSMIC Gene Id (COSG), COSMIC Sample Id (COSS), COSMIC Structural Id (COST), COSMIC CNV Id (COSCNV), COSMIC Gene Fusion Id (COSF), Legacy Mutation Id (COSM/COSN), COSMIC Paper Id (COSP), COSMIC Study Id (COSU), COSMIC Genomic Mutation Id (COSV). Furthermore, each download file is now packaged with a read-me file, while a consistent file name convention helps users to better navigate between the genome assemblies, product releases and to construct their analytic pipelines.

### New resources

*Mutational Signatures catalogue v3.3* (https://cancer.sanger.ac.uk/signatures/) is a resource developed as part of the CRUK Mutographs Cancer Grand Challenge and a collaborative project involving the Wellcome Sanger Institute, the Pillay lab at University College London, and the Alexandrov lab at the University of California (17). Mutational signatures are patterns of somatic mutations associated with specific mutagenic processes. They can be considered a way of identifying various types of damage within the genome that have accumulated prior to, and during the development and progression, of a cancer. A mutational signature of a cancer can be informative with regard to the nature of the likely source of DNA damage (e.g. UV exposure, smoking, exposure to specific treatments etc.), or be indicative of inactivation of one of the DNA damage repair pathways. For example mutational signatures are used to detect the deficiency of the homologous recombination pathway in cancer samples, indicative for therapy with PARP inhibitors (18).

COSMIC’s mutational signature catalogue has been built through the analysis of the PCAWG dataset (2) using the SigProfiler algorithm (17) and additional curation of selected published studies, where data originate from samples exposed to various DNA-altering factors. Currently, four different mutational signature classes are considered, resulting in a set of 155 individual mutational signatures: SBS (single base substitution signatures; *n* = 95), DBS (doublet base substitution signatures; *n* = 11), ID (insertion deletion signatures; *n* = 18), CN (copy number signatures; *n* = 21). Within these four classes, each reference signature is described, acknowledging its validation status, proposed aetiology and tissue distribution, potential associations with other mutational signatures and various genomic features, and how the signature has changed compared to the previous versions of the resource.

A recently added feature, the SigProfiler Assignment web tool (19) (https://cancer.sanger.ac.uk/signatures/assignment/) allows users to upload their own sequenced samples in VCF or MAF format to assign the reference mutational signatures to these samples, utilising a custom implementation of the forward stagewise algorithm and non-negative least squares.

*The Cancer Mutation Census* (https://cancer.sanger.ac.uk/cmc/home) integrates biological, biochemical and population information from multiple sources, allowing users to discover and understand which mutations can drive different types of cancer. It is aimed at improving the application of precision oncology and to help users understand which mutations are likely to be causing cancer, and which are only a result of the disease – passenger mutations.

To assess the oncogenic potential of each of over 5 million coding mutations in COSMIC, manually curated information regarding cancer genes obtained from the Cancer Gene Census and genetic variants, acquired from ClinVar (20) is integrated with data on variant frequencies across multiple cancer types from the COSMIC catalogue. For each variant, this is supported by information on its germline frequency in normal populations (gnomAD) (21), signs of positive selection in cancer cells (dN/dS) (22), as well as conservation on DNA (GERP) (23) and protein (SIFT) (24) level. A simple and transparent set of rules is applied to identify variants with the highest potential of clinical relevance. The CMC website (https://cancer.sanger.ac.uk/cmc) presents the distribution of all CMC mutations, their characteristics and properties within each gene helping to identify clusters of significant mutations, presence and distribution of SNPs in that gene, or assessing conservation of certain fragments of the gene compared to others.

All the data in the CMC are downloadable and interpretations are fully transparent to provide clarity as to why a mutation is classified with either high or low impact. Users are able to see the biological reasoning and the evidence base used.

*The scope of COSMIC Actionability* is all interventional oncology clinical trials or case studies that have either selected patients by the presence of a variant, or state the intention to correlate efficacy with the presence of a variant. Studies that require the patient to express a protein target or compare efficacy between patients expressing at differing levels are also included. To date (actionability v10), clinical data has been curated for 988 actionable variants, including 156 point mutations, in 445 genes. A total of 11121 trials are represented with 1873 treatments, in 5174 treatment combinations across 226 cancer types.

All Actionability records indicate the source of the data and aim to represent the most complete and recent trial results. As clinical trials rarely identify variants at the DNA level, variants in Actionability are identified at the protein level. Actionability content is curated by gene; a gene is considered fully curated when every relevant trial identified by systematic manual searching in clinical trial repositories and the scientific literature has been recorded. Content for fully curated genes is updated at every release and all previously-recorded trials are re-examined for results updates and new trials, initiated since the previous release.

### Resources with updated and expanded content

*The Cancer Gene Census* (CGC) (7) (https://cancer.sanger.ac.uk/census) is a catalogue of genes affected by somatic alteration and/or rare pathogenic germline mutation that contribute to cancer development. The CGC is compiled by manual curation of peer-reviewed, published mutation data and experimental evidence demonstrating that a gene has a function relevant to cancer. For each gene, the CGC details the cancer types most frequently affected by somatic alteration and/or rare pathogenic germline mutation, the types of somatic alteration affecting the gene in cancer, and the role, or roles, of the gene in one, or more, cancer types. In COSMIC release v98, the CGC includes 738 genes (compared to 719 in v86), partitioned into two tiers (Tier 1: 579 genes; Tier 2: 159 genes) based on the strength of the evidence that associate them with cancer. Each gene is manually evaluated for the presence of somatic mutation patterns typical for cancer drivers (e.g. hotspots of missense or inframe indel mutations characterising oncogenes) and evidence of molecular function associated with potential to drive hallmarks of cancer. For a gene to be qualified to Tier 1, both types of evidence must be present in at least two independent sources. Tier 2 of the CGC comprises genes with convincing evidence of their involvement in cancer development that do not fully fulfil all the requirements for Tier 1 qualification.

In COSMIC release v98, Hallmarks of Cancer annotations are provided for 352 out of 579 CGC Tier 1 genes (92 additional genes characterised since v86). The Hallmarks of Cancer are the phenotypic characteristics shared by cancers (25–27). In COSMIC, these disease-level traits have been adapted to provide a means of describing how genes functionally contribute to cancer development. To achieve that, available publications are studied by a curator to identify gene's involvement in cellular processes that participate in generation of the hallmarks of cancer, such as control of cell division, conducting proliferative signalling, or triggering apoptosis. This often requires performing independent interpretations of experimental results and includes specifying if the function of the protein has potential to drive or suppress each hallmark. Every ‘Hallmark gene page’ provides graphical and tabular summaries of which cancer hallmarks are affected by a wild type protein. Up to 22 data fields collectively constitute a cancer-associated fully referenced mini-gene review. These annotations include summaries of gene function, somatic alterations (including gene fusions) and high penetrance germline mutations that affect the gene in cancer, the impact of coding mutations on protein function, and clinically-relevant gene attributes. New Hallmark gene pages are added to every COSMIC release.

*The catalogue of somatic mutations causing drug resistance*. COSMIC annotates mutations identified in the literature as conferring resistance to treatment, specifically the mutations acquired after the treatment. In the case of solid tumours, tumours from patients that have received therapy and reported a drug response are annotated in COSMIC using a Drug Response Tumour Feature, based on RECIST (Response Evaluation Criteria in Solid Tumours) (28) terms. Acquired resistance mutations occur in tumours annotated as having ‘resistant recurrence’ drug response phrase. Those acquired mutations reported to be associated with resistance, or presumed by authors to be associated with resistance, are annotated as secondary resistance mutations and included in COSMIC’s catalogue.

Through the export files COSMIC v98 provides information curated from 2945 patient samples, describing 476 mutations in 39 genes responsible for secondary resistance to 60 drugs in 111 cancer types.

*The Cancer Browser* (https://cancer.sanger.ac.uk/cosmic/browse/tissue) allows website users to query the database from a disease-specific start point, allowing for selection and filtering of COSMIC data by tissue type and histology, i.e. cancer type. Mutation frequencies and profiles within selected disease specific subsets can be explored. The cancer browser lists 49 primary tissue types at the first level of selection in the tissue browser. With subsequent refinements for sub-tissue, histology and sub-histology, the browser provides insights into the somatic alterations of 4161 cancer types. The most frequently mutated genes are graphically displayed for the selected subset. Each gene can then be further explored by displaying its mutation profile within that subset.

*COSMIC-3D* (29) (https://cancer.sanger.ac.uk/cosmic3d) integrates cancer mutations with protein structure data across the human genome and structural proteome, showcasing over 53 000 experimentally determined protein structures with interactive 3D visualisations to help understand the impact of somatic mutations in the context of protein 3D structure. The resource is updated with new mutations and newly available PDB protein structures at every COSMIC release.

## Access to COSMIC

### Websites

All COSMIC resources are accessible through the main COSMIC web portal (https://cancer.sanger.ac.uk), which offers summaries and visualisations of data, providing gene profiles including mutation frequencies across 4161 cancer types, detailed nature of mutations, and where available, functional consequence of gene dysfunction via Hallmarks of Cancer, gene expression (*z* scores) and methylation data. The likely functional significance and clinical actionability of specific mutations can be explored separately through the Cancer Mutation Census and Actionability websites, respectively.

### Downloads

For power users, the full content of the COSMIC knowledgebase is available through a collection of export files. Data from the core COSMIC database, the Cancer Gene Census, Cancer Mutation Census, Cell Lines Project, Actionability, as well as definitions of mutational signatures are all available for download; as COSMIC-3D represents data from COSMIC in new visual forms, no downloadable content is provided. All data referring to a human genome are available for both GRCh37 and GRCh38 genome assemblies. The core coding mutation content is available in a tabular form as well as files in vcf format, and other types of genetic alterations, such as structural variants, non-coding variants, gene fusions, gene expression variants, differential DNA methylation, or resistance mutations are provided within specific files.

Genome wide datasets for download can be found on the download page. https://cancer.sanger.ac.uk/cosmic/download for COSMIC, Cancer Gene Census, Cancer Mutation Census, Cell Lines Project, and Actionability data, or https://cancer.sanger.ac.uk/signatures/downloads/ for the mutational signatures’ definitions.

### Licensing

To download COSMIC data and use certain functionalities of the websites (CMC), users must register for a COSMIC account. COSMIC is available for free to non-commercial users, whilst a licence fee is applicable to commercial users to support continued growth and maintenance of the resource. Both commercial and non-commercial users have full access to all the data for each new COSMIC release.

## Summary

COSMIC’s role in the global cancer research ecosystem is helping the research community conduct their science better and faster by delivering biologically and clinically-relevant genomic knowledge across all cancer types. Through provision of sustainable and accessible bioinformatic datasets and software COSMIC also aims to bridge academic and patient-specific applied sciences. Integrating, standardising and adding highly curated annotation to the data enhances its value and provides a knowledge base to drive current and future research. As an integral part of the research community, COSMIC actively addresses many common challenges faced by those involved in genomic research. These are important factors that will determine the future development of COSMIC and similar resources. One prominent issue is the lack of diversity in genomic datasets. Differences in variant prevalence between global populations are a well-known phenomenon in the germline variant research (30) but in certain cases also patterns of somatic mutations differ between patients with the same cancer type but different ethnic origin (31). Translating these genetic differences into effective clinical practice requires more comprehensive and unbiased genomic datasets. Therefore, an effort has to be made to maximise ethnic and phenotypic diversity in public datasets. Additionally, the absence of widely accepted standards for reporting, processing, and sharing genomic data hinders valuable scientific and clinical information from entering the public domain. These challenges, however, are all addressable. The COSMIC team remains committed to collaborating with scientists and clinicians to overcome these obstacles, thereby fostering the continued development of resources like COSMIC for the betterment of cancer research and patient care.

## Supplementary Material

gkad986_supplemental_file

## Data Availability

All COSMIC resources are accessible through the main COSMIC web portal (https://cancer.sanger.ac.uk).
