# Supplementary material for: COSMIC: a curated database of somatic variants and clinical data for cancer
Source: Nucleic Acids Res. 2023 Nov 1;52(D1):D1210–7. doi: 10.1093/nar/gkad986 (PMC10767972; doi:10.1093/nar/gkad986)
Supplement: gkad986_supplemental_file [file gkad986_supplemental_file.pdf]

GLOSSARY

**Systematic screens**	Studies covering substantial fraction of the genome, reporting somatic variants mapped to genomic coordinates in a tabular format.
**Targeted screens**	Studies providing sequencing data for a subset of genes as opposed to indiscriminate whole exome or whole genome screening. Often, information about particular variants is spread across the different sections of the publication, its figures, and supplementary data.
**VEP**	Variant Effect Predictor is Ensembl tool used by COSMIC to annotate genetic variants, i.e. map them to the reference genome assemblies for further classification into different variant types.
**Data**	In the context of COSMIC curation, a unit of curated information referring to a genetic variant and a cancer type.
**Metadata**	Data providing information about one or more aspects of the COSMIC data. For the core COSMIC curation, it includes ethnicity, age, drug response, therapy, cytogenetics, tumour grade and stage, and others.
**HGVS**	Human Genome Variation Society nomenclature for standardised description of sequence variants in DNA, RNA, and protein sequences.
**Somatic mutations**	mutations (variants) that arise in somatic cells. Somatic mutations are not inherited by the offspring.
